# The safety of sports in children with inherited arrhythmia substrates

**DOI:** 10.3389/fped.2023.1151286

**Published:** 2023-04-04

**Authors:** Abhay Katyal, Christopher O. Y. Li, Sonia Franciosi, Shubhayan Sanatani

**Affiliations:** British Columbia Children’s Hospital Heart Center, Department of Pediatrics, University of British Columbia, Vancouver, BC, Canada

**Keywords:** sports, exercise, physical activity, sudden cardiac death, channelopathy, cardiomyopathy, implantable cardioverter-defibrillator, pediatrics

## Abstract

Sudden cardiac death (SCD) is a rare and devastating event in children and remains a leading cause of death in young athletes. Channelopathies and cardiomyopathies, in particular long QT syndrome (LQTS), catecholaminergic polymorphic ventricular tachycardia (CPVT), hypertrophic cardiomyopathy (HCM), and arrhythmogenic cardiomyopathy (ACM) are associated with exercise-related SCD. Implantable cardioverter-defibrillators (ICDs) are often placed for secondary prevention for athletes with cardiomyopathy or channelopathy. There remains concern regarding the safety of return to participation with an ICD in place. Guidelines have historically recommended that patients with inherited heart rhythm disorders be restricted from competitive sports participation. Increasing evidence suggests a lower risk of exercise-related cardiac events in young athletes with inherited heart rhythm disorders. In this review, we highlight current knowledge, evolving guidelines, and present a multidisciplinary approach involving shared decision-making and appropriate planning for safe sports participation of children with inherited heart rhythm disorders.

## Introduction

Sudden cardiac death (SCD) is a rare and devastating event in children and remains a leading cause of death in athletes aged 40 years or younger. Estimates show the incidence of SCD in young individuals (typically aged under 35–40 years) to be between 1 and 3 cases/100,000 (1). Exercise-related SCD is frequently caused by inherited heart rhythm conditions: channelopathies and cardiomyopathies. In particular long QT syndrome (LQTS), catecholaminergic polymorphic ventricular tachycardia (CPVT), hypertrophic cardiomyopathy (HCM), and arrhythmogenic cardiomyopathy (ACM) are associated with exertional ventricular arrhythmias. Guidelines recommend implantable cardioverter-defibrillators (ICDs) for secondary prevention in these conditions (2). Concern has been raised around sports participation for children with ICDs (3). Previous guidelines have recommended against sports participation for children with inherited heart rhythm disorders and ICDs due to an abundance of caution and fear of cardiac events (4).

Christian et al. reported that approximately half of their cohort of pediatric patients with either LQTS, CPVT, ACM, or HCM had reduced quality of life and modified their physical activity after receiving their diagnosis (5). Patients advised against physical activity because of their condition are at risk of gaining weight and obesity (5). Exercise restriction in children with inherited heart rhythm disorders can also lead to physical deconditioning and additional cardiovascular burden later in life (6). Reineck et al. found that the psychosocial and emotional well-being of HCM patients was negatively impacted due to exercise restriction (7).

**Figure 1:**
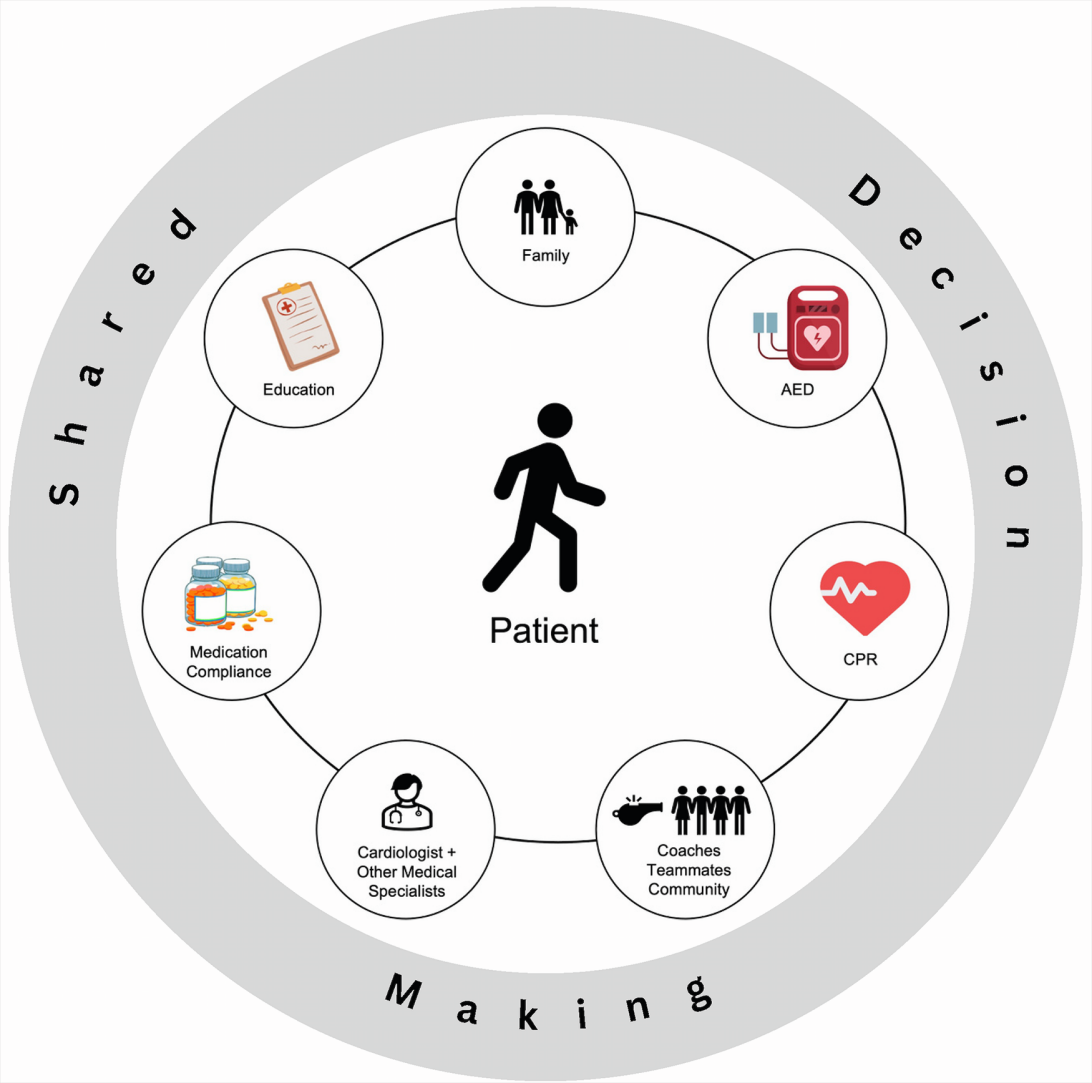
Shared decision-making model for sports participation. CPR, cardiopulmonary resuscitation; AED, automated external defibrillator.

Patients with inherited heart rhythm disorders are advised to modify their physical activity due to the perceived risk of exercise-related cardiac events. There is evidence that children with inherited heart rhythm disorders are at a lower risk of exercise-related cardiac events than previously thought (6, 8). Optimized medical management and creation of a safety plan are necessary to promote exercise participation for children affected by channelopathies and cardiomyopathies. The field is moving away from exercise restriction and towards shared decision-making, which places the patient at the center of the discussion (Figure 1). Appropriate risk assessment, regular expert follow-up, including a review of treatment compliance, increasing community awareness, and access to an automated external defibrillator (AED) are all important components to ensuring safe participation. In this review, we summarize current knowledge, guidelines, and present our multidisciplinary approach with appropriate planning in place so that pediatric patients with inherited heart rhythm disorders can participate in sports safely.

## Cardiac ion channelopathies & sports participation

### Sports participation guidelines for channelopathies

In 2005, guidelines on exercise recommendations and sports participation were published by the European Society of Cardiology (ESC) and an expert task force from the 36th Bethesda Conference (4, 9). The American Heart Association (AHA), American College of Cardiology (ACC), and ESC subsequently published a report on the management of ventricular arrhythmias and prevention of SCD (10). These guidelines advised against competitive sports participation for inherited arrhythmias and were mostly based on expert consensus with low levels of evidence. Guidelines define a competitive athlete as an individual who participates in regular exercise training and competes in organized individual or team sports (9, 11, 12). This definition encompasses a wide range of ages as athletes can compete at various levels of competition (i.e., youth, high-school, college, national). Each guideline recommendation is categorized by a class of recommendation and level of evidence (LOE) (Table 1) (13, 14). Class I refers to strong recommendations where the benefits outweigh the risks. Class IIa (moderate) and IIb (weak) recommendations have some degree of benefit to the population. Class III reflects recommendations that either have no benefit or cause harm. LOE A is high quality evidence derived from multiple randomized-controlled trials or meta-analyses. LOE B is moderate quality evidence from other randomized or non-randomized studies that have not been externally validated. LOE C is given to guidelines based on either low quality of evidence or consensus of expert opinion. An international consensus statement published in 2013 from the Heart Rhythm Society (HRS), European Heart Rhythm Association (EHRA), and Asia Pacific Heart Rhythm Society (APHRS) on the diagnosis and management of patients with inherited primary arrhythmia syndromes followed a similar approach (15).

**Table 1:** Levels of evidence.

Level of Evidence A	Data derived from multiple randomized clinical trials or meta-analyses.
Level of Evidence B	Data derived from a single randomized clinical trial or large non-randomized studies.
Level of Evidence C	Consensus of opinion of the experts and/or small studies, retrospective studies, registries.

© ESC 2020 Pellicia et al., 2020 ESC Guidelines on sports cardiology and exercise in patients with cardiovascular disease, Eur Heart J, 2021, 42, 1, 17–96, DOI: 10.1093/eurheartj/ehaa605. Reprinted by permission of Oxford University Press on behalf of the European Society of Cardiology.

The 2015 AHA guidelines provided recommendations on sports participation for patients with cardiac ion channelopathies and presented new strategies for clinical management (16). Experts suggested that athletes should first be evaluated comprehensively by a heart rhythm specialist or genetic cardiologist (Class I; LOE C). If symptomatic, athletes should be restricted from participating in competitive sports until both they and their family are well informed, a treatment plan is in place, and the athlete has been asymptomatic after adhering to their therapy for more than three months (Class I; LOE C). They recommended that children can be considered for sports participation if they are asymptomatic or genotype-positive/phenotype-negative, and if certain precautionary measures are in place (e.g., having a personal AED and avoidance of dehydration/electrolyte imbalance) (Class IIa; LOE C). The 2020 and 2022 ESC guidelines remained stringent with their approach with few updates from their 2005 guidelines and recommended that symptomatic athletes be restricted from all competitive sports (12, 17). Specific recommendations for each channelopathy are outlined in each section below.

### Long QT syndrome

Long QT syndrome (LQTS) is an inherited channelopathy characterized by a prolonged QT interval and T-wave abnormalities on the electrocardiogram (ECG). Approximately 1 in 2000 people are affected by LQTS, making it the most common cardiac channelopathy (18). The majority of patients have loss of function variants affecting the repolarizing potassium channels. The prolonged repolarization can lead to a characteristic type of ventricular tachycardia (VT) known as torsade de pointes, which causes syncope, sudden cardiac arrest (18), and SCD. Annual rates of syncope and SCD in untreated patients are 0.33%–0.9% and 5%, respectively (19). To date, mutations in 17 genes associated with LQTS have been found (20). The supporting data for many of these mutations is limited to small pedigrees and populations. The three most commonly implicated genes are *KCNQ1*, *KCNH2*, and *SCN5A*, and pathogenic variants in these three genes are responsible for 75%–80% of all congenital LQTS (21). β-blocker therapy remains the mainstay of treatment. Other interventions include avoidance of QT prolonging medications (www.crediblemeds.org), implantable cardioverter-defibrillators (ICDs) and left cardiac sympathetic denervation (LCSD).

In the past, children with LQTS had been advised against sports participation in accordance with guidelines and due to the perceived higher risk of recurrent cardiac events. Early data showed that exercise-related cardiac events in patients with LQTS were most commonly linked to the LQT1 genotype (62%), followed by LQT2 (13%) and LQT3 (13%) (18). LQT1 patients were observed to be at a higher risk of having cardiac events when exercising, especially during participation in activities involving high adrenergic stress (22). Swimming is a specific trigger in LQTS1(22). They observed that patients with LQT2 and LQT3 genotypes were more likely to experience cardiac events during rest/sleep (29% and 39%) compared to when exercising (13%). Auditory and emotional triggers of cardiac events were more prevalent among LQT2 patients (26%) compared to those with the LQT1 and LQT3 genotypes (2% and 7%).

#### What the guidelines say about LQTS

Previous guidelines recommended against competitive sports participation for LQTS patients (4, 9, 10). Guidelines from the 2005 36th Bethesda Conference advised that all patients with LQTS should be restricted from competitive sports, especially swimming, with the exception of minimal contact class 1A sports (e.g., billiards, bowling, cricket, curling, golf, and riflery) (4). In 2013, an HRS/EHRA/APHRS expert consensus statement modified their perspective for low-risk LQTS patients (15). Experts recommended that non-LQT1 genotyped asymptomatic patients with borderline QTc prolongation and no family history of SCD could be considered for competitive sports participation after a thorough clinical evaluation (Class I Recommendation). The 2015 AHA guidelines recommended that LQTS athletes who are symptomatic or have a baseline QT prolongation (QTc > 470 milliseconds in males and >480 milliseconds in females) could potentially participate in sports, except for competitive swimming in the LQT1 athlete (Class IIb; LOE C) (16). They further stated this would only be considered if they are asymptomatic on treatment for at least three months, not taking QT prolonging drugs, and follow appropriate precautionary measures. The 2020 ESC guidelines remained consistent with their previous recommendations in that high-intensity recreational and competitive sports participation should be avoided by LQTS patients, even when on β-blocker therapy (Class III; LOE B) (2, 12). Recent 2022 ESC guidelines recommended that LQTS patients avoid QT-prolonging drugs, avoid and correct electrolyte abnormalities, and avoid genotype- specific triggers for arrhythmias as described above (17).

#### Current LQTS sports participation evidence

Evidence supporting return to play for children with LQTS has grown significantly. In 2013, Johnson and Ackerman determined the outcomes of LQT1–3 patients who chose to continue to participate in competitive sports despite guideline recommendations (23). From a cohort of 353 patients, 130 (37%) participated in competitive sports, including 20 with ICDs. There were no LQT-triggered cardiac events among competitive athletes in over 650 patient-years. Medication compliance was implicated in two breakthrough cardiac events that occurred while preparing to compete in sporting events. In 2015, Aziz et al. conducted a retrospective review of genotype-positive LQTS pediatric patients to determine prevalence and outcomes of sports participation (24). Of the 212 patients who received β-blocker therapy, 26 (26%) participated in competitive sports and 77 (75%) participated in recreational sports. There was non-compliance and intolerance to β-blockade treatment in two patients. No patients experienced LQTS symptoms or cardiac events while participating in sports. These results highlight the importance of medication compliance.

Recent data further supports the safety of sports participation in LQTS patients. Chambers et al. reported no cardiac events or deaths in 106 athletes who participated in competitive sports (25). Four patients who were previously symptomatic with very prolonged QT intervals had cardiac events during recreational exercise. Tobert et al. reviewed the electronic medical records of 494 LQTS patients and reported no exercise-related deaths with 2056 combined years of follow-up (21). Three athletes (0.6%) experienced a cardiac event related to sports participation. In 2022, our center reported that physical activity levels were similar in 23 LQTS pediatric patients compared to healthy controls (26). These studies also support the safety of a shared decision-making model.

### Catecholaminergic polymorphic ventricular tachycardia

Catecholaminergic polymorphic ventricular tachycardia (CPVT) is a primary electrical disease characterized by bidirectional and polymorphic VT during adrenergic stress, normal resting ECG, and a structurally normal heart (27). It is a rare condition estimated to affect 1 in 10,000 individuals (15). An individual with CPVT may experience palpitations, light-headedness, syncope, and sudden cardiac arrest (SCA)/SCD during physical activity or emotional stress. CPVT is caused by abnormal calcium handling in the cardiomyocyte. This is most commonly caused by gain-of-function variants in the cardiac ryanodine receptor-2 (*RYR2*) gene and is inherited in an autosomal dominant manner. CPVT can also be caused by genetic variants in the genes encoding calsequestrin-2 (*CASQ2*) and calmodulin (*CALM1*) (28).

The current treatment strategies for CPVT include β-blockers as the first-line treatment, with flecainide, LCSD, and ICDs as additional therapies (29). Flecainide has been increasingly used in treating CPVT in the past decade (30, 31). The correct medication and dosage, along with ensuring medication compliance is important in managing CPVT.

#### What the guidelines say about CPVT

The 2005 36th Bethesda Conference guidelines recommended that all patients with CPVT should be restricted from competitive sports, especially swimming, with the exception of minimal contact class 1A sports (4). Experts indicated that a less restrictive approach may be considered for genotype-positive/phenotype-negative athletes. The 2013 HRS/EHRA/APHRS international consensus statement and 2015 ESC guidelines recommended that CPVT patients limit or avoid competitive sports, strenuous exercise, and stressful environments (Class I; LOE C) (2, 15). The 2015 AHA guidelines suggested against competitive sports participation for previously symptomatic or asymptomatic CPVT athletes if they have exercise-induced premature ventricular contractions (PVCs) in bigeminy, couplets, or non-sustained VT (Class III; LOE C) (2, 16). Although the 2015 AHA guidelines advised disqualification, they stated that the athlete/family could potentially consider sports participation after a thorough consultation with a CPVT specialist (16). The 2022 ESC guidelines recommended that patients with CPVT should avoid competitive sports, strenuous exercise, and stressful environments (Class I; LOE C) (17).

#### Current CPVT sports participation evidence

Current evidence challenges exercise restriction in CPVT patients. The risk of recurrent cardiac events is lower than reported in early series, especially during exercise (32, 33). In fact, exercise may be beneficial in CPVT. In 2012, Kurtzwald-Josefson et al. found that exercise training can improve cardiac function and reduce arrhythmia in *CASQ2* knockout mice that have a similar phenotype to human CPVT2 (34). Faster supraventricular rates induced with atropine can prevent ventricular arrhythmias, bidirectional VT, and ventricular ectopic beats during exercise in CPVT mice and patients (35, 36).

Ostby et al. conducted a retrospective study to investigate the outcomes in CPVT patients who were non-athletes (*n* = 43) or continued to participate in sports (*n* = 21) (37). Eight of 21 (38%) patients competed competitively as college athletes or adults. Only three athletes (14%) had cardiac breakthrough events: two were associated with recreational exercise and one was due to non-compliance with β-blocker therapy. Six non-athletes (14%) who were symptomatic had CPVT-linked cardiac events, and two non-athletes did not comply with their β-blocker treatment. This further underscores the importance of medication compliance in preventing events.

## Cardiomyopathies & sports participation

### Sports participation guidelines for cardiomyopathies

Due to the risk of SCA/SCD during sports and exercise particularly in young patients with cardiomyopathies, guidelines were developed to reduce risk through disease specific management and activity restriction. Previous guidelines advised routine disqualification of athletes diagnosed with cardiomyopathies from competitive sports (9, 38). ACC/AHA & European Association of Preventive Cardiology (EAPC) guidelines have shifted to a more case-by-case basis with a shared decision-making approach involving the patient, family members, and physician/cardiologists (12, 39, 40). Coaches should be informed of the patient's condition to ensure ongoing surveillance including potential occurrence of SCA during sports participation as well as to ensure that an emergency action is in place if a SCA were to occur (38). Return to play has not been well studied in young competitive athletes with cardiomyopathies.

### Arrhythmogenic cardiomyopathy

ACM is an inherited structural heart disorder that predisposes individuals to ventricular arrhythmias and SCA. It typically presents between the second and fourth decade of life. In ACM, risk of arrhythmias and disease progression is accelerated by exercise (41). It presents more often as SCA/SCD in pediatric patients than in adults (42, 43). Structural changes are characterized by an enlarged, dilated right/left ventricle with or without decreased systolic function. There is fibro-fatty degeneration of the right ventricular (RV) (5) and/or left ventricular (LV) (44) myocardium which is associated with frequent arrhythmias. Most cases are inherited in an autosomal dominant manner, and due to causative variants in genes encoding the desmosome. During early stages of the disease, there is a subclinical phase where structural abnormalities are concealed and patients exhibit no symptoms. Patients are still at risk of experiencing SCA/SCD during this phase (45). This concealed phase then progresses to an electrical phase characterized by abnormalities on ECG including T-wave inversions, PVCs, and VT with left bundle branch block morphology. The structural phase presents with phenotypic alterations including right or biventricular dilatation and potentially heart failure.

Task force criteria may underestimate incidence of ACM in children (46). The ECG is insensitive in detecting ACM in young asymptomatic patients (47). Characteristics on ECG, PVC burden, complex PVC, cardiac magnetic resonance (CMR) results are important for diagnosis and determining disease progression (43, 45). Further family history, genetic testing, multimodal imaging and reassessment as the child grows are crucial in order to determine diagnosis and treatment (48). A risk model for ACM encompassing age, sex, syncope, nonsustained VT, PVCs, T-wave inversions on ECG, and RV ejection fraction has been developed for adult patients, but this has not been validated yet in pediatric patients (13).

#### What the guidelines say about ACM

The 2005 ESC guidelines recommended against participation in competitive sports for ACM patients (9). An ACC/AHA 2015 expert task force advised that ACM patients should not participate in most competitive sports, with the possible exception of class 1A sports (Class III; LOE C) (38). In 2015, ESC experts recommended avoidance of competitive sports, remaining stringent with their approach as there were no controlled trials showing beneficial effects of exercise in ACM patients (Class I; LOE C) (2). Since 2017, both national and international guidelines have suggested that ACM patients should avoid competitive or high-intensity endurance exercise as they are linked to increased risk of ventricular arrhythmias and disease progression (12, 49, 50). The 2020 ESC guidelines recommended that ACM patients should participate in 150-minute low intensity exercise per week (Class IIa; LOE C) (12). Experts stated that participation in low-to-moderate intensity recreational exercise/sports may be considered in patients with no history of cardiac arrest and minimal structural abnormalities (Class IIb; LOE C). The 2022 ESC guidelines recommended against participation in high-intensity/competitive sports for ACM patients including those who are genotype-positive/phenotype-negative (Class IIb, LOE C) (17). Gow et al. demonstrated that children with inherited arrhythmia conditions routinely exceed recommended activity levels (51). Evidence of disease progression and worse outcomes in 80 subjects with the *TMEM43* p.S358L ACM mutation who participated in high-intensity exercise [>9 metabolic equivalent of task (MET)-hours/day] led to the recommendation that cascade screening be done earlier, to inform exercise choices reflective of the genotype (52). Cascade screening is a systematic approach of identifying family members of a proband who may be at risk for an inherited arrhythmia condition *via* clinical screening and genetic testing (53).

#### Current ACM sports participation evidence

Exposure to exercise accelerates ACM disease progression and reducing exercise can lower the risk of experiencing ventricular arrhythmias (41). Pediatric-onset ACM has been associated with increased endurance activity compared to adult-onset disease (42). Lie et al. showed that adult ACM patients were more likely to experience ventricular arrhythmias if they participated in high-intensity exercise (74%) and long-duration exercise (65%) (54). Phenotype-negative desmosomal gene-positive females who participated in high-intensity and long-duration exercise during adolescence were at greater risk of developing ACM (55). Wang et al. found that ACM patients with primary prevention ICDs and those who were gene-elusive benefited from exercise reduction (56). Adult *TMEM43* positive ACM patients who participated in high-intensity exercise in the year prior to ICD implantation experienced a 9-fold-increase in appropriate ICD discharge compared to patients who participated in moderate-intensity exercise (52). Half of previously athletic ACM patients still experienced ventricular arrhythmias despite exercise reduction, therefore suggesting that ICDs should continue to be recommended if high-risk features exist (56). Smith et al. found that desmoplakin associated cardiomyopathy showed no correlation between exercise and risk of ventricular arrhythmias or LV/RV dysfunction, indicating that risk of arrhythmias may be specific to genotype (57). Risk and outcomes in ACM can change over time, therefore, it is imperative that ACM patients engaged in low or moderate activity be assessed yearly (12, 58).

Adolescents who are genotype-positive/phenotype-negative should be potentially restricted from high intensity sports and counselled about possible long term risks (48). Activities such as soccer, aerobics, or fast competitive swimming on a regular basis should be avoided. Restriction from competitive sports participation is regarded as an important preventative tool for both asymptomatic (gene-positive) and symptomatic patients as it can slow disease progression and prevent life threatening arrhythmias (48). To date, there has been no compelling data supporting the safety of exercise in ACM; in particular, data examining pediatric patients is lacking.

### Hypertrophic cardiomyopathy

HCM is characterized by an abnormally thickened ventricular myocardium that can manifest at any age (59). There are a number of causes, particularly in infants and children, in whom inborn errors of metabolism and syndromic causes predominate (60). In adults, the disease is caused by variants in genes encoding the thick and thin contractile myofilament protein components of the sarcomere or adjacent Z discs. Inheritance is autosomal dominant with variable penetrance. The HCM phenotype varies from asymptomatic to LV outflow tract obstruction, diastolic dysfunction, heart failure, tachyarrhythmias and SCA/SCD. Although the presence of LV hypertrophy is considered a diagnostic hallmark of the disease, arrhythmia, SCA/SCD may present prior to structural changes (61). Clinical evaluation should include family history, echocardiogram, ECG, Holter monitoring and exercise stress test to determine risk for sudden death. CMR with late gadolinium imaging should be considered in addition to echocardiography to determine the scar burden (62). The phenotype can progress as the child grows. Models for SCD risk prediction in pediatric HCM have been developed (63, 64). The risk models include predictors such as age, maximal LV wall thickness, left atrial diameter, LV outflow tract gradient, family history of SCD, non-sustained VT, and unexplained syncope. LV outflow tract obstruction can increase during exercise and ischemia may result. HCM pathology is associated with myocyte disarray and ischemia. In the young, the extent of HCM pathology was found to be greater than older patients and correlated with an abnormal vascular response to exercise (65).

#### What the guidelines say about HCM

The 2005 ESC guidelines stated that physical activity could induce hypertrophy and recommended against competitive sports participation for HCM patients (9). Guidelines from the 36th Bethesda conference indicated that HCM athletes may be at a greater risk for SCA/SCD during exercise (4). The 2014 ESC guidelines on the diagnosis and management of HCM advised against participation in competitive sports and intense physical activity (66). The 2015 ACC/AHA guidelines restricted patients with a probable HCM diagnosis from participating in competitive sports, with the possible exception of class 1A sports (Class III; LOE C) (38). Experts stated competitive sports participation may be considered for asymptomatic genotype-positive HCM patients without evidence of LV hypertrophy, especially if they have no family history of HCM-related sudden death (Class IIa; LOE C). More recent 2020 ACC/AHA and ESC guidelines have shifted to the perspective that high-intensity recreational activities or moderate-to high-intensity competitive sports may be considered after a comprehensive evaluation and shared decision-making approach (12, 39, 40).

#### Current HCM sports participation evidence

HCM is a frequent cause of SCD in young competitive athletes (67). In a HCM mouse model, exercise before development of the phenotype prevented myocyte fibrosis and other markers of myocardial hypertrophy. Exercise reversed myocyte disarray and other markers of hypertrophy in older mice (68). In 1998, Corrado et al. reported 49 sudden deaths within a cohort of 33,735 competitive athletes (mean age of 19 years) who underwent pre-participation screening (69). 22 athletes who had definite evidence of HCM were disqualified from sports participation and no mortality was reported during follow-up. This data indicated that pre-participation screening in detecting HCM can be beneficial in reducing mortality. Malhotra et al. screened 11,168 adolescent soccer players (mean age of 16 years) and 5 were identified to have HCM (70). Over a follow-up period of 10.6 years, 3 of the 5 patients stopped to play and survived whereas two players continued to play and died.

Training early in young HCM patients may be beneficial in preserving LV diastolic function later in life (71). Greater exercise training during childhood and adolescence in HCM phenotype-positive or genotype-positive patients correlated with more favorable LV relaxation, filling, and end diastolic LV volume (72). Young HCM patients who participated in competitive sports have also been found to have a larger LV cavity size and better diastolic function compared to sedentary HCM patients (73). Exercise can reduce blood pressure (74). As hypertension accelerates HCM and is known to be a major cause of LV hypertrophy, exercise could be beneficial in limiting adverse hypertensive effects (75–77). Among 31 low-risk athletes with HCM, there was no difference in incidence of symptoms or major cardiac events between athletes who stopped exercise and those who continued to participate in competitive sports (78). Athletes were classified as either high-risk or low-risk according to risk factor profile scores set by SCD algorithms developed by the AHA and ESC (79). An HCM athlete is considered as low-risk if they have less than four of the following risk factors for SCD: non-sustained VT, severe LV hypertrophy, family history of SCD, unexplained syncope, and abnormal blood pressure response to exercise. Pelliccia et al. conducted a follow-up study with low-risk HCM phenotype patients (19–44 years old) who either continued to participate in competitive sports or reduced or stopped exercise (80). Over a follow-up period of 7 years, there was no difference in risk of SCA/SCD between HCM-trained and detrained patients. Therefore, in low-risk HCM patients, participation in competitive sports does not appear to increase risk of cardiac arrest or death. These results may not reflect those with a more severe HCM phenotype or younger adolescent HCM patients who are greater risk of SCD.

## Safety of sports for young patients with ICDS

ICDs are recommended for most patients following cardiac arrest for secondary prevention of SCA. In some cases, ICDs for primary prevention are implanted in those considered at high arrhythmic risk. The risk-benefit balance of primary prevention ICDs in the pediatric population are not well established. However, ICDs in young patients with inherited heart rhythm disorders are associated with a longer-term risk of inappropriate shocks and device-related complications (81). While life-saving, adjustment to life with an ICD can be challenging (82). Risk factors associated with poor coping after ICD implantation include young age and frequent shocks (83).

### What the guidelines say about ICDs

Prior to 2015, experts recommended against competitive sports participation for children with ICDs due to higher perceived risks as there could be potential failure of the ICD to defibrillate, risk of death due to ICD shock, or damage to the device. After initial supporting results from the ICD Sports Safety Registry, the 2015 ACC/AHA guidelines stated that competitive sports participation could be considered for athletes with ICDs (16, 84). Expert consensus indicates that the decision whether a young patient with an ICD participates in sports should be patient specific (44). The underlying cardiac disease, type of device, age, and activity needs to be considered. ICD implantation is not advised in asymptomatic patients with a diagnosis of LQTS or CPVT (Class III; LOE C). If β-blockade treatment is ineffective or intolerable for symptomatic LQTS patients and other interventions (e.g., LCSD) are not effective, then ICDs are recommended (Class I; LOE B). ICDs are indicated for symptomatic CPVT patients who have experienced cardiac arrest or refractory ventricular arrhythmias despite being on maximally tolerated combination medical therapy (Class I; LOE C). ICDs are recommended for ACM patients with sustained VT, syncope associated to ventricular arrhythmia, or an LV ejection fraction ≤35% (Class I; LOE B). In HCM patients with primary risk factors (e.g., LV hypertrophy, unexplained syncope), ICDs are recommended after considering the complications that can arise given the disease progression.

ICDs should not be implanted for the purpose of sports participation (Class III; LOE B) (44). Once an athlete with an ICD returns to play, medications and device settings should be optimized. Programming the ICD with high-rate cut-offs and long-detection duration in athletes can reduce inappropriate shocks without affecting survival (85). Further, an emergency action plan, as we discuss in further detail below, consisting of informed and cardiopulmonary resuscitation (CPR) trained coaches and staff, quick action plans, and availability of an AED is crucial for safe return to play (86).

### ICDs and current sports participation evidence

The multinational ICD Sports Safety Registry was established to assess the risk of sports participation for athletes with ICDs (84). 328 athletes (10–60 years old) with ICDs participating in competitive sports were recruited. Results showed that there were no occurrences of death, resuscitated arrhythmia, or shock-related injury during sports participation (84, 87). Saarel et al. conducted a subanalysis of the registry and collected data on sports and clinical outcomes from a cohort of 129 young athletes (10–21 years old) (3). These athletes were followed over a median of 3.5 years, and the most common diagnoses were LQTS, HCM, and congenital heart disease. Results indicated that young patients with ICDs can participate in competitive and high-intensity sports without failure of ICDs to terminate arrhythmias or injury (3). Sports participation was not associated with significant adverse events, although appropriate and inappropriate shocks did occur (3, 84, 87).

## Multidisciplinary approach & safe sports participation recommendations

It is important to discuss activity participation upon making the diagnosis of an inherited heart rhythm condition. There are a lot of misconceptions, and the tendency is to restrict activity without necessarily enabling participation. It is important that patients understand what their diagnosis is, and the specifics of that condition. Over the past three decades, causal genes implicated in inherited heart rhythm disorders have been successfully identified, and this has played a significant role in how these diseases are diagnosed and managed today. This means that pre-symptomatic patients and those without a phenotype are being diagnosed. The importance of genetic counselling in probands with inherited arrhythmias and family members has become widely recognized. Confronting patients with the risk of SCD and the possibility of transmitting the potentially fatal disease to their relatives may result in adverse psychological consequences; this distress highlights the importance of psychological support for these patients (88).

The patient and physician should engage in shared decision-making when discussing exercise participation (Figure 1). Shared decision-making considers patient goals and preferences balanced against the risk and benefits of exercise using clear communication. This discussion should include the most current evidence, nature of the athletic activity, sport-specific risks, and individual athlete characteristics and goals, all to arrive at patient-centered treatment decisions. It is important to bear in mind that current exercise guidelines are not exclusions in absolute terms. Almost all patients with inherited heart rhythm conditions can and should participate in exercise. It is important that patients find the most suitable exercise for their condition and lifestyle. Our starting point is that there are no exercises or sports that are not open for discussion. Rather, the focus is encouraging the patient to participate if they can develop a feasible safety plan for the activity. It is important to promote activity, not just remove restriction. The exception to this approach may be ACM, in which prolonged and intense exercise may accelerate the disease progression. It is also important to emphasize that prolonged and highly intense exercises are not required for patients to obtain cardiovascular benefits.

**Figure 2:**
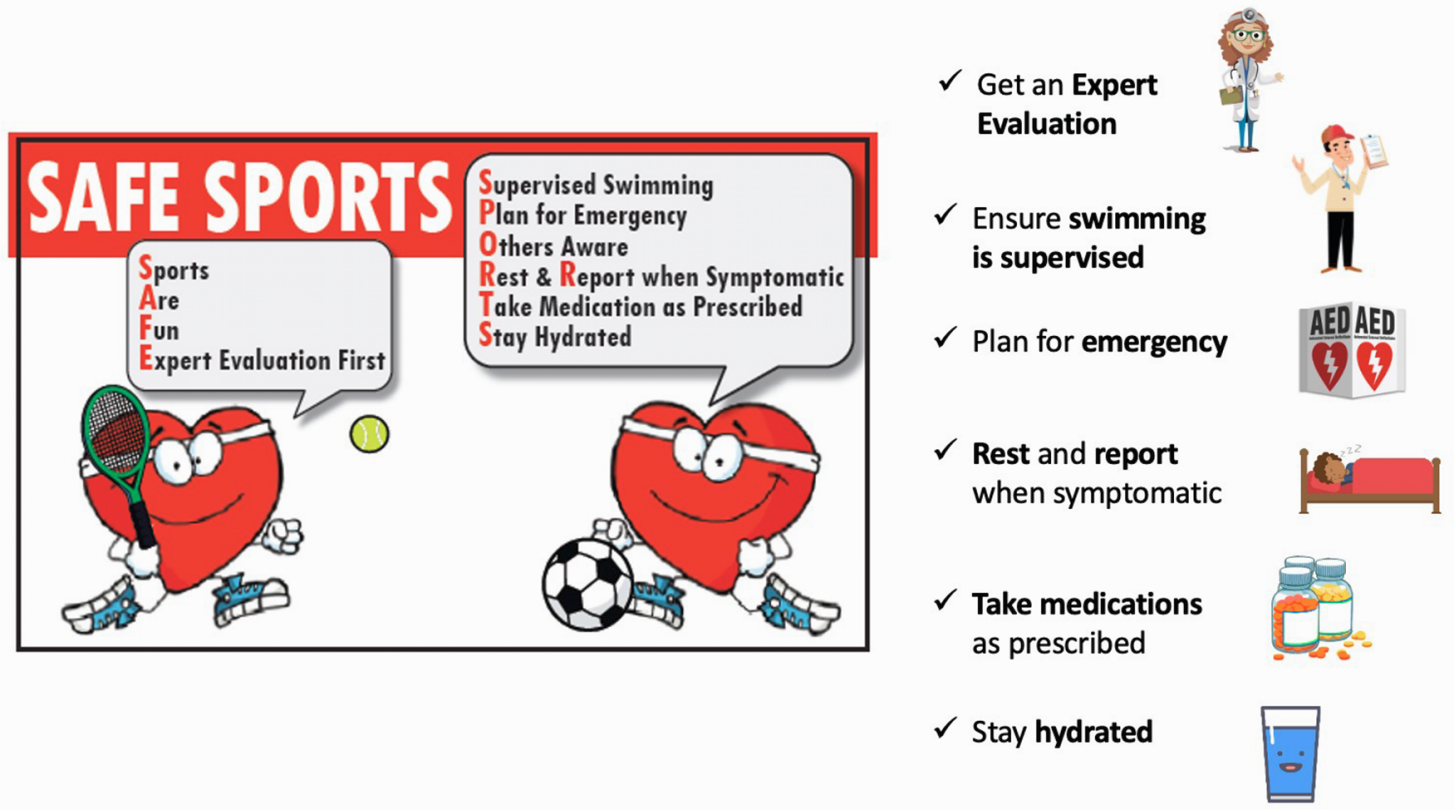
Safety of sports in children with inherited heart rhythm disorders. Modified from Cheung et al., 2016 (89).

Our knowledge and the patient's condition can change over time, and therefore patients should attend regular appointments with their healthcare providers. Those choosing to participate in exercise should be made aware of benign and potentially serious cardiac symptoms that may occur during activity. Patients should rest if they notice these symptoms and contact their healthcare provider for further evaluation (Figure 2). Medication compliance is of utmost importance. Strenuous activity in hot environments, dehydration, and poor nutrition preceding activity can lead to electrolyte disturbances; adequate hydration and nutrition are always advised prior to exercise participation.

The inherited heart rhythm clinic should support the athlete by providing clear communication to families, coaches, trainers, and schools. Athletes and those around them including family, teammates, coaches, trainers, and school staff should have a plan in place in the event of a potential SCA. This includes having protocols in place to notify first responders, knowing where to retrieve the AED, and educating personnel in CPR and AED use (90). Team sports or exercises with peers are preferred in the event that help is required. Coaches and schools can incorporate AEDs into team equipment. AEDs should be available and rapidly accessible at home, in the community, and at competitions. Training for this life-saving skill is easy to access through many different local organizations.

## Conclusions

Sports participation in children with inherited heart rhythm disorders has been widely debated by experts in the field, with a tendency to restrict activities extensively. Exercise restriction can have a negative impact on the child's overall quality of life and well-being. Current data where available have not supported the benefit of such restrictions. Activity can be safely promoted in all conditions with perhaps the exception of ACM. A multidisciplinary approach that includes shared decision-making with appropriate risk assessment and follow-up, and development of safety plans can lead to healthier lifestyles. Increased community awareness regarding CPR and AED use are important measures for the rare breakthrough events.
